# Chemical Composition of the Oleogum Resin Essential Oils of *Boswellia dalzielii* from Burkina Faso

**DOI:** 10.3390/plants8070223

**Published:** 2019-07-14

**Authors:** Anjanette DeCarlo, Stephen Johnson, Amadé Ouédraogo, Noura S. Dosoky, William N. Setzer

**Affiliations:** 1 Aromatic Plant Research Center, 230 N 1200 E, Suite 100, Lehi, UT 84043, USA; 2 Laboratory of Plant Biology and Ecology, University Joseph Ki-Zerbo, 03 BP 7021 Ouagadougou 03, Burkina Faso; 3 Department of Chemistry, University of Alabama in Huntsville, Huntsville, AL 35899, USA

**Keywords:** frankincense, olibanum, essential oil composition, α-pinene

## Abstract

Frankincense, the oleogum resin from members of *Boswellia*, has been used as medicine and incense for thousands of years, and essential oils derived from frankincense are important articles of commerce today. A new source of frankincense resin, *Boswellia dalzielii* from West Africa has been presented as a new, alternative source of frankincense. In this work, the oleogum resins from 20 different *Boswellia dalzielii* trees growing in Burkina Faso, West Africa were collected. Hydrodistillation of the resins gave essential oils that were analyzed by GC-MS and GC-FID. The essential oils were dominated by α-pinene (21.0%–56.0%), followed by carvone (2.1%–5.4%) and α-copaene (1.8%–5.0%). Interestingly, there was one individual tree that, although rich in α-pinene (21.0%), also had a substantial concentration of myrcene (19.2%) and α-thujene (9.8%). In conclusion, the oleogum resin essential oil compositions of *B. dalzielii*, rich in α-pinene, are comparable in composition to other frankincense essential oils, including *B. sacra*, *B. carteri*, and *B. frereana*. Additionally, the differences in composition between samples from Burkina Faso and those from Nigeria are very slight. There is, however, a rare chemotype of *B. dalzielii* that is dominated by myrcene, found both in Burkina Faso as well as Nigeria.

## 1. Introduction

Frankincense is an aromatic oleoresin with a volatile fraction typically composed primarily of terpenoids and more rarely ethers or fatty esters/alcohols [1]. The oleoresin is produced by the 20 members of the genus *Boswellia* (Burseraceae: Sapindales), which are distributed across sub-Saharan Africa, Arabia, and the Indian subcontinent [1,2,3]. In nature, the oleoresins defend the trees against infection and pests such as boring beetles, while humans have used them for up to 5000 years for medicine and incense [4]. Today, the oleoresins of many species are traded internationally and distilled into essential oil for aromatherapy and perfumery. The oleoresin essential oils have been characterized for most of the *Boswellia* genus, with the exceptions of *B. microphylla* Chivo., *B. ogadensis* Vollesen, and *B. globosa* Thulin [5,6,7,8,9,10,11,12,13,14,15,16,17,18,19,20,21].

**Figure 1:**
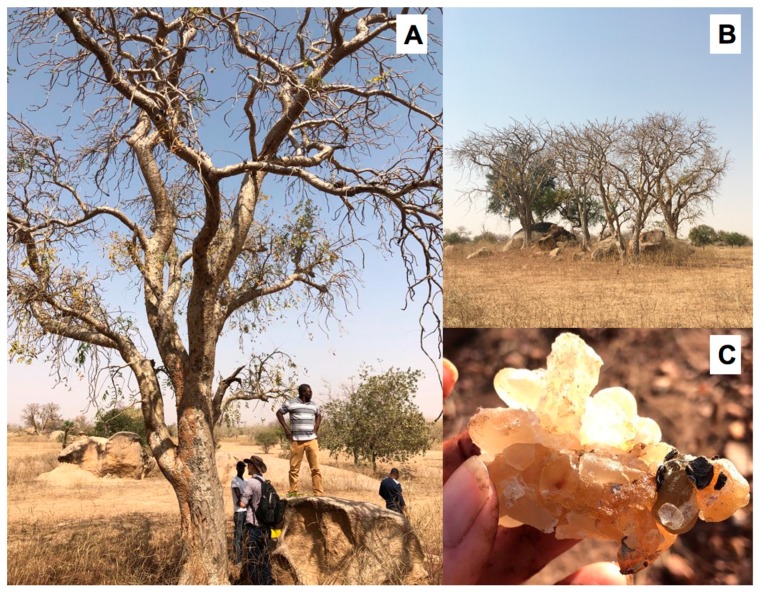
*Boswellia dalzielii* tree in situ (**A**); Grove of *B. dalzielii* trees (**B**); Oleogum resin from self-exuded by a *B. dalzielii* tree (**C**).

Despite being one of the most wide-ranging species, the oleoresin essential oil of *Boswellia dalzielii* has only recently been examined [21]. *Boswellia dalzielii* (see Figure 1) inhabits wooded to open savannahs from Chad to Mali; the most significant populations appear to be in Burkina Faso, Nigeria, and Mali. The trees are 4–13 m tall, typically with papery or scaly bark, fragrant white flowers, serrated compound leaves, and aromatic resin [22]. Although resin harvesting has only recently increased, the bark is harvested extensively for its use in tinctures to treat malaria, toothaches, sores, and snakebites [23,24,25,26,27,28].

Although there have been a number of studies on the compounds in the bark, there has been little work on the volatile compounds in this species. Two studies have examined the hydrodistilled leaf essential oils of *B. dalzielii*; Kohoude et al. found that the oil was dominated by δ-3-carene (27.7%) and α-pinene (15.2%) with smaller amounts of *p*-cymene (9.5%), β-phellandrene (8.5%), isolongifolene (6.2%), and myrcene (5.7%) [29], while Kubmarawa et al. found oils dominated by α-pinene (45.7%) and α-terpinene (11.5%) [30]. Recent work by DeCarlo et al. provided the first study on *B. dalzielii* oleoresin essential oil, examining single-tree oleoresin samples from northern Nigeria [21]. They found that the majority of essential oils were dominated by α-pinene (21.7%–76.6%), some with lower levels of α-thujene (2.0%–17.6%) and *p*-cymene (0.3%–15.6%); a second, much rarer chemotype was rich in myrcene (up to 35.2%), sometimes with a significant level of limonene (up to 32.9%). These samples were found to be rich in monterpenes but almost devoid of sesquiterpenes.

Along with Nigeria, Burkina Faso hosts one of the largest populations of *B. dalzielii*. The species is widespread across three of the four phytogeographic zones of the country (from 500 to 900 mm annual rainfall range). Populations are gregarious and colonize more often rocky hills and glacis. Mean density of trees in natural stands ranges between 7 and 10 trees per 1000 m^2^ according to phytogeographic zones in Burkina Faso [31]. The regeneration is very poor due to human disturbances (bushfire, pasture, agriculture) and climate pejoration (drought, rain fluctuations) [32,33].

The prior work on *B. dalzielii* oleoresin essential oil from Nigeria found multiple chemotypes as well as intrachemotypic compositional variation [21], and several other *Boswellia* species are known to display multiple chemotypes [1,5,11,19] (unpublished results from our laboratory). Given the extensive geographical range of *B. dalzielii*, it’s likely that additional chemical diversity is present beyond that captured in the study from Nigeria. Therefore, in this study, we examine the essential oils from oleoresins taken directly from individual trees in Burkina Faso to determine if additional chemical variation is present. Each oleoresin sample was hydrodistilled using the same apparatus (Clevenger) and analyzed by GC-MS and GC-FID by the same operators under the same conditions.

## 2. Results

Essential oils were obtained by hydrodistillation of the *B. dalzielii* oleogum resin samples in yields of 1.69%–17.0% (*v*/*w*) as pale-yellow essential oils. The chemical compositions of the essential oils are compiled in Table 1. Nineteen of the twenty samples were dominated by α-pinene (26.3%–56.0%), with minor levels of α-copaene (1.8%–5.0%), carvone (2.1%–5.0%), bornyl acetate (1.6%–3.5%), α-cubebene (1.2%–3.4%), myrcene (0.4%–5.5%), α-thujene (0.5%–9.2%), and γ-terpinene (0.8%–2.6%). One sample contained almost equal levels of myrcene (19.2%) and α-pinene (21.0%), with moderate α-thujene (9.8%) and α-copaene (3.8%).

**Figure 2:**
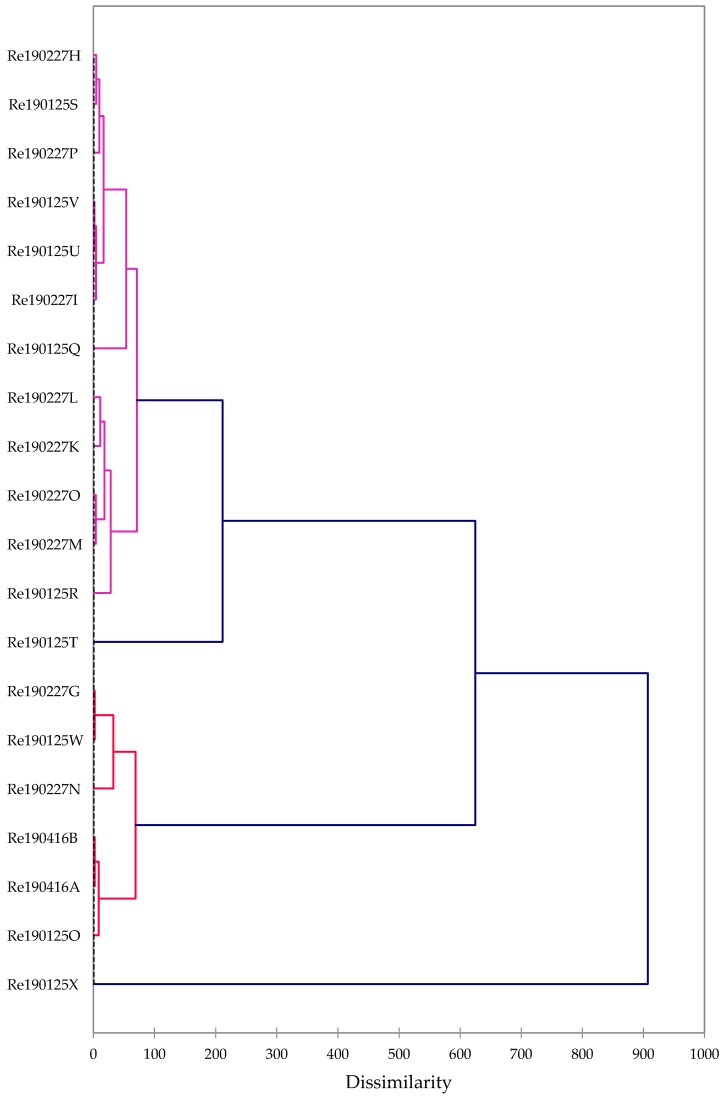
Dendrogram obtained from the agglomerative hierarchical cluster analysis of 20 *Boswellia dalzielii* oleogum resin essential oil compositions from Burkina Faso.

A hierarchical cluster analysis of the essential oil compositions revealed two major groups (Figure 2): One dominated by high α-pinene, and one rich in myrcene. Although unusual, the myrcene sample is only moderately dissimilar to the predominant α-pinene samples; we therefore conclude that there is a single, α-pinene dominant chemotype with a rare subchemotype rich in myrcene. The chemical compositions do not appear to correlate with the geographical locations from which they were taken, either within central Burkina Faso or between central and western Burkina Faso.

## 3. Discussion

The oleogum resin essential oil of *Boswellia dalzielii* has only been described once previously, to our knowledge, in oleoresins taken from trees in northern Nigeria [21]. Our results are largely consistent with the findings of that study: In both areas, the essential oils are most commonly dominated by α-pinene, with a small number of samples showing high levels of myrcene. Levels of α-pinene were generally higher in Nigeria (42.6%–72.1%) compared to Burkina Faso (21.0%–56.0%) and α-thujene and *p*-cymene were less prevalent in this study. Additionally, in contrast to the samples from Nigeria, the samples from this study were found to contain an appreciable percentage of sesquiterpenes, particularly α-copaene.

The leaf essential oils of *B. dalzielii* have also been observed to contain a significant proportion of α-pinene, although other major components in those oils such as δ-3-carene, α-terpinene, and *p*-cymene were only observed in minor quantities in the oleoresin essential oils [29,30]. *Boswellia dalzielii* is rich in monoterpenes, particularly α-pinene, similar to many *Boswellia* species; *Boswellia sacra* and *Boswellia carteri* oleoresin essential oils are most commonly dominated by α-pinene, as well as lesser amounts of α-thujene, limonene, myrcene, sabinene, and *p*-cymene [11,13,18,34,35,36]. Many *B. frereana* essential oils are similarly dominated by α-pinene, with lower levels of sabinene and *p*-cymene [7,13,19]. A second chemotype of *B. frereana* is dominated by α-thujene, as are the oleoresins of *B. serrata* from India and *B. ameero*, *B. dioscoridis*, *B. elongata*, *B. nana*, and *B. popoviana* from Socotra Island, Yemen [12,17,37,38] (unpublished results from our laboratory). By contrast, several *Boswellia* species have completely different chemical profiles: *B. papyrifera* essential oils are dominated by octyl acetate and to a lesser degree octanol [8,13,39]; *B. occulta* oils have methoxyalkanes as the major components [11,40,41]; and *B. bullata* produces an unusual mix of δ-cadinene, β-caryophyllene, (*E*)-β-farnesene, α-cadinol, and several unidentified components [37]. The *Boswellia dalzielii* oleoresin essential oils are therefore fairly similar to those of several commonly traded commercial species (*B. frereana*, *B. sacra*, *B. carteri*).

Further work will be necessary to determine if the essential oils from Burkina Faso versus Nigerian *B. dalzielii* oleoresins show differential biological activities. In general, the reason for the diversity of terpenes and especially the importance of minor constituents is not clear [42]. The differential profiles likely convey some ecological benefits, though, as even essential oils from the same species, showing only modest chemical differences, do vary significantly in the degree to which they inhibit different strains of microbial pathogens [34]. This may indicate that the variation is related to variation in pathogenic threats.

The myrcene-dominated resins are intriguing as they represent only a small number of samples and differ greatly from the dominant α-pinene chemotype in both Nigeria and Burkina Faso. Environmental conditions can influence the chemical composition of plant volatiles [43,44,45]; although there was no geographic pattern observed here with regard to chemical variation, the myrcene chemotype samples taken in Nigeria were from the same geographic location. However, biotic factors also play a role: in a study of the heartwood essential oil of *Santalum insulare* in the Marquesas Islands, different chemotypes were observed in trees only a few meters apart, implying differences based on genetics or possibly pathogen attack history [46]; our laboratory has also observed different oleoresin chemical compositions in *Boswellia carteri* trees only a few meters apart in Somaliland (unpublished results from our laboratory). Further work is thus necessary to elucidate the reasons for the observed chemotypic differences in *B. dalzielii*.

## 4. Materials and Methods

### 4.1. Collection of Oleogum Resins

**Figure 3:**
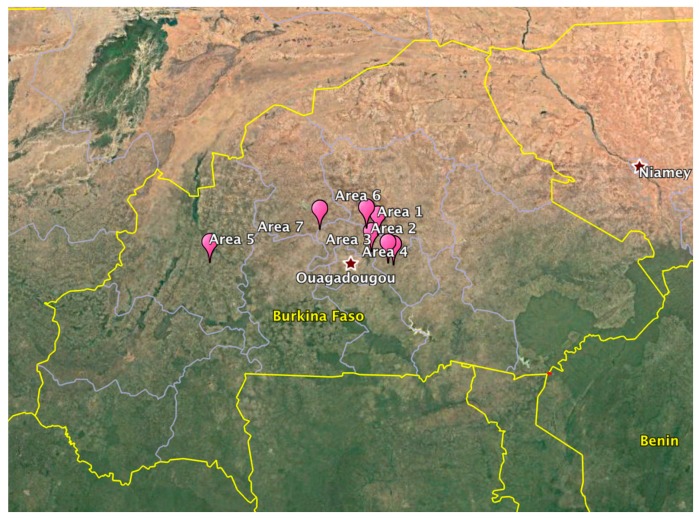
Locations where *B. dalzielii* oleogum resin samples were collected.

Twenty *Boswellia dalzielii* oleoresin samples were collected directly from source trees in six locations across central and western Burkina Faso (Figure 3). Samples were collected during the dry season (February–March 2019), and each sample location was GPS tagged (Table 2). All samples were taken from fresh resin exuding either naturally from tree branches or from wounds left by recent bark harvesting; consequently, age differences in the resin were not enough to significantly alter the resins′ chemical compositions. The trees were identified in the field by Anjanette DeCarlo and Stephen Johnson. A voucher specimen (Voucher number OUA6892) was deposited at the University of Ouagadougou herbarium and the identification confirmed by Amadé Ouédraogo, a botanist working there.

### 4.2. Hydrodistillation of Oleogum Resins

Hydrodistillations of the *Boswellia dalzielii* oleoresin samples were carried out in an all-glass Clevenger-type apparatus as previously described [21].

### 4.3. Gas-Chromatographic-Mass Spectral Analysis

Each of the *B. dalzielii* oleogum resin essential oils was analyzed by GC-MS as previously described [21]: Shimadzu GCMS-QP2010 Ultra (Shimadzu Scientific Instruments, Columbia, MD, USA), ZB-5ms capillary column (Phenomenex, Torrance, CA, USA). Identification of the essential oil components was based on their retention indices determined by reference to a homologous series of *n*-alkanes, and by comparison of their mass spectral fragmentation patterns with those reported in the literature [47], and our in-house library.

### 4.4. Gas Chromatographic-Flame Ionization Detection

Analysis of the *B. dalzielii* oleogum resin essential oils by GC-FID was carried out as previously described [21]: Shimadzu GC 2010 with flame ionization detector (Shimadzu Scientific Instruments, Columbia, MD, USA), ZB-5 capillary column (Phenomenex, Torrance, CA, USA). The percent compositions listed in Table 1 are averages from three separate runs of the essential oils were determined from peak areas and corrected using response factors for the different classes of chemical components [48].

### 4.5. Hierarchical Cluster Analysis

The chemical compositions of the *B. dalzielii* oleoresin essential oils were used in the hierarchical cluster analysis. The 20 essential oil compositions were treated as operational taxonomic units (OTUs), and the concentrations (percentages) of 23 major components were used to determine the chemical associations between these frankincense essential oils using agglomerative hierarchical cluster (AHC) analysis using XLSTAT Premium, version 2018.5.53172 (Addinsoft, Paris, France). Dissimilarity was determined using Euclidean distance, and clustering was defined using Ward’s method.

## 5. Conclusions

*Boswellia dalzielii* oleoresin essential oils from Burkina Faso are similar to those from Nigeria in that they are generally dominated by α-pinene, but unlike the samples from Nigeria, those in the current study contained a significant percentage of sesquiterpenes. The reason for these differences is not clear, but it potentially points to different biological and ecological activity due to different pathogenic threats. A rare chemotype or subchemotype dominated by myrcene was observed both in this study and in samples from Nigeria; this is potentially also related to specific pathogenic threats that may exist in both locations, or possibly genetic factors. This species thus offers a promising option for future work to elucidate the drivers of intraspecific chemical variation.

## Figures and Tables

**Table 1:** Chemical compositions of *Boswellia dalzielii* oleogum resin essential oils from Burkina Faso.

**RI a**	**RI b**	**Compound**	**Re190227M**	**Re190125O**	**Re190125Q**	**Re190125R**	**Re190125S**	**Re190125T**	**Re190125U**	**Re190125V**	**Re190125W**	**Re190125X**
**906**	**906**	**Santolina triene c**	**1.2 ± 0.0 d**	**1.0 ± 0.0**	**1.0 ± 0.0**	**1.8 ± 0.0**	**1.2 ± 0.0**	**1.5 ± 0.0**	**1.1 ± 0.0**	**1.0 ± 0.0**	**1.0 ± 0.0**	**1.3 ± 0.0**
**918**	**919**	**5,5-Dimethyl-1-vinylbicyclo [2.1.1] hexane**	**1.1 ± 0.0**	**1.1 ± 0.0**	**0.6 ± 0.0**	**1.0 ± 0.0**	**1.1 ± 0.0**	**1.0 ± 0.0**	**1.4 ± 0.0**	**0.9 ± 0.0**	**0.7 ± 0.0**	**1.5 ± 0.0**
920	921	Tricyclene	0.2 ± 0.0	0.2 ± 0.0	0.1 ± 0.0	0.1 ± 0.0	0.2 ± 0.0	0.2 ± 0.0	0.1 ± 0.0	0.2 ± 0.0	0.2 ± 0.0	0.1 ± 0.0
**923**	**924**	**α-Thujene**	**1.4 ± 0.0**	**3.1 ± 0.0**	**9.2 ± 0.0**	**0.7 ± 0.0**	**3.0 ± 0.2**	**5.0 ± 0.1**	**0.7 ± 0.0**	**1.1 ± 0.0**	**0.5 ± 0.0**	**9.8 ± 0.2**
**932**	**932**	**α-Pinene**	**39.6 ± 1.5**	**56.0 ± 0.0**	**41.3 ± 0.1**	**34.9 ± 0.6**	**43.3 ± 2.3**	**26.3 ± 0.3**	**44.1 ± 0.6**	**43.5 ± 0.1**	**49.7 ± 0.0**	**21.0 ± 0.4**
945	945	α-Fenchene	tr e	tr	tr	---	tr	tr	tr	tr	tr	tr
**947**	**946**	**Camphene**	**0.9 ± 0.0**	**1.1 ± 0.0**	**0.8 ± 0.0**	**0.8 ± 0.0**	**1.3 ± 0.1**	**1.1 ± 0.0**	**0.7 ± 0.0**	**1.0 ± 0.0**	**0.8 ± 0.0**	**0.8 ± 0.0**
951	953	Thuja-2, 4(10)-diene	0.8 ± 0.0	0.7 ± 0.0	0.6 ± 0.0	1.0 ± 0.0	0.8 ± 0.0	1.0 ± 0.0	0.7 ± 0.0	0.6 ± 0.0	0.4 ± 0.0	0.8 ± 0.0
953	954	β-Fenchene	0.2 ± 0.0	0.4 ± 0.0	0.1 ± 0.0	0.5 ± 0.0	0.1 ± 0.0	0.1 ± 0.0	0.2 ± 0.0	0.1 ± 0.0	0.2 ± 0.0	tr
**970**	**969**	**Sabinene**	**1.3 ± 0.0**	**1.3 ± 0.0**	**1.5 ± 0.0**	**1.1 ± 0.0**	**1.2 ± 0.0**	**1.4 ± 0.0**	**0.9 ± 0.0**	**0.1 ± 0.0**	**0.8 ± 0.0**	**1.9 ± 0.0**
**975**	**974**	**β-Pinene**	**1.4 ± 0.0**	**2.0 ± 0.0**	**1.6 ± 0.0**	**0.8 ± 0.0**	**1.5 ± 0.1**	**1.4 ± 0.0**	**1.7 ± 0.0**	**1.3 ± 0.0**	**1.4 ± 0.0**	**1.1 ± 0.0**
985	984	*trans-p*-Mentha-2, 8-diene	---	---	---	---	---	---	---	---	---	---
**986**	**988**	**Myrcene**	**1.0 ± 0.0**	**0.9 ± 0.0**	**0.5 ± 0.0**	**0.9 ± 0.0**	**0.8 ± 0.1**	**1.1 ± 0.0**	**1.6 ± 0.1**	**1.0 ± 0.0**	**0.4 ± 0.0**	**19.2 ± 0.3**
988	989	3, 3, 7-Trimethylcyclohepta-1, 3, 5-triene	0.1 ± 0.0	tr	tr	0.2 ± 0.0	---	tr	---	0.1 ± 0.0	tr	---
996	997	(*E*)-2,6-Dimethyl-2, 6-octadiene	---	---	---	---	---	---	---	---	---	---
998	1001	δ-2-Carene	---	---	---	---	---	---	---	---	---	---
999	995	*cis-p*-Menth-8-ene	0.7 ± 0.0	0.9 ± 0.0	0.6 ± 0.0	0.8 ± 0.0	0.6 ± 0.0	0.8 ± 0.0	0.7 ± 0.0	0.6 ± 0.0	0.5 ± 0.0	0.4 ± 0.0
1002	1003	*p*-Mentha-1(7), 8-diene	---	---	---	---	---	---	---	---	---	tr
1003	1002	α-Phellandrene	tr	0.1 ± 0.0	tr	---	tr	tr	tr	tr	0.1 ± 0.0	---
1004	1006	1,5,8-*p*-Menthatriene	0.4 ± 0.0	0.4 ± 0.0	0.4 ± 0.0	0.4 ± 0.0	0.3 ± 0.0	0.4 ± 0.0	0.3 ± 0.0	0.4 ± 0.0	0.2 ± 0.0	0.7 ± 0.0
1006	1005	*o*-Cresol methyl ether	tr	0.1 ± 0.0	0.1 ± 0.0	0.1 ± 0.0	0.1 ± 0.0	tr	0.1 ± 0.0	0.1 ± 0.0	---	tr
1006	1008	δ-3-Carene	---	0.2 ± 0.0	tr	0.2 ± 0.0	tr	tr	tr	tr	0.1 ± 0.0	0.1 ± 0.0
1015	1014	α-Terpinene	0.5 ± 0.0	0.6 ± 0.0	0.5 ± 0.0	0.6 ± 0.0	0.4 ± 0.0	0.5 ± 0.0	0.4 ± 0.0	0.5 ± 0.0	0.3 ± 0.0	0.5 ± 0.0
1017	1022	*m*-Cymene	0.6 ± 0.0	0.9 ± 0.0	0.6 ± 0.0	0.7 ± 0.0	0.7 ± 0.0	0.7 ± 0.0	0.6 ± 0.0	0.6 ± 0.0	0.3 ± 0.0	0.5 ± 0.0
1021	1021	*p*-Menth-1-ene	---	---	---	---	---	---	---	---	tr	tr
1022	1024	*p*-Cymene	0.5 ± 0.0	1.2 ± 0.0	0.7 ± 0.0	0.6 ± 0.0	1.0 ± 0.0	1.1 ± 0.0	0.3 ± 0.0	1.1 ± 0.0	0.3 ± 0.0	0.4 ± 0.0
1024	1026	2-Acetyl-5-methylfuran	0.1 ± 0.0	0.1 ± 0.0	0.3 ± 0.0	0.4 ± 0.0	0.2 ± 0.0	0.4 ± 0.0	tr	0.1 ± 0.0	tr	tr
1027	1024	Limonene	0.2 ± 0.0	0.3 ± 0.0	0.3 ± 0.0	0.3 ± 0.0	0.3 ± 0.0	0.3 ± 0.0	0.2 ± 0.0	tr	0.1 ± 0.0	0.2 ± 0.0
1028	1025	β-Phellandrene	tr	0.1 ± 0.0	0.2 ± 0.0	0.1 ± 0.0	tr	tr	tr	0.1 ± 0.0	0.1 ± 0.0	0.4 ± 0.0
**1029**	**1026**	**1,8-Cineole**	**0.9 ± 0.0**	**1.8 ± 0.0**	**1.0 ± 0.0**	**0.6 ± 0.0**	**0.9 ± 0.0**	**0.9 ± 0.0**	**0.6 ± 0.0**	**1.0 ± 0.0**	**0.9 ± 0.0**	**1.3 ± 0.0**
1031	1032	(*Z*)-β-Ocimene	0.2 ± 0.0	0.3 ± 0.0	0.2 ± 0.0	0.2 ± 0.0	0.2 ± 0.0	0.2 ± 0.0	0.3 ± 0.0	0.3 ± 0.0	0.1 ± 0.0	0.1 ± 0.0
**1032**	**1039**	***o*-Cymene**	**1.2 ± 0.0**	**2.0 ± 0.0**	**1.1 ± 0.0**	**1.5 ± 0.0**	**1.1 ± 0.1**	**1.4 ± 0.0**	**1.2 ± 0.0**	**1.1 ± 0.0**	**1.0 ± 0.0**	**1.5 ± 0.0**
1043	1044	(*E*)-β-Ocimene	0.1 ± 0.0	0.3 ± 0.0	0.1 ± 0.0	---	0.1 ± 0.0	tr	0.1 ± 0.0	0.1 ± 0.0	0.1 ± 0.0	0.3 ± 0.0
**1055**	**1054**	**γ-Terpinene**	**1.9 ± 0.0**	**0.8 ± 0.0**	**2.0 ± 0.0**	**2.0 ± 0.1**	**1.9 ± 0.1**	**2.6 ± 0.0**	**1.8 ± 0.0**	**2.0 ± 0.0**	**1.5 ± 0.0**	**1.9 ± 0.0**
1068	1065	*cis*-Sabinene hydrate	tr	tr	tr	0.1	tr	0.1 ± 0.0	tr	tr	tr	tr
**1083**	**1086**	**Terpinolene**	**1.2 ± 0.0**	**0.5 ± 0.0**	**1.1 ± 0.0**	**1.5 ± 0.0**	**1.3 ± 0.1**	**1.3 ± 0.0**	**1.4 ± 0.0**	**1.5 ± 0.0**	**1.0 ± 0.0**	**1.3 ± 0.0**
1088	1089	*p*-Cymenene	0.1 ± 0.0	---	---	---	---	---	0.1 ± 0.0	0.1 ± 0.0	---	---
1088	1090	6,7-Epoxymyrcene	---	---	---	---	---	---	---	---	---	tr
1090	1095	6-Camphenone	---	---	---	---	---	---	---	---	---	---
1094	1091	Rosefuran	tr	---	---	---	---	---	---	tr	---	---
1096	1102	Perillene	tr	tr	---	---	---	---	0.1 ± 0.0	tr	---	tr
1097	1095	Linalool	1.3 ± 0.0	0.6 ± 0.0	1.0 ± 0.0	1.4 ± 0.0	1.2 ± 0.0	1.8 ± 0.0	1.3 ± 0.0	1.0 ± 0.0	1.0 ± 0.0	1.1 ± 0.0
**1097**	**1099**	**α-Pinene oxide**	**tr**	**0.2 ± 0.0**	**0.1 ± 0.0**	**0.5 ± 0.0**	**0.7 ± 0.0**	**0.2 ± 0.0**	**0.4 ± 0.0**	**0.1 ± 0.0**	**0.1 ± 0.0**	**---**
1099	1098	*trans*-Sabinene hydrate	---	---	tr	0.1 ± 0.0	---	tr	---	tr	tr	0.1 ± 0.0
**1103**	**1101**	***cis*-Thujone**	**1.2 ± 0.0**	**0.6 ± 0.0**	**1.2 ± 0.0**	**1.4 ± 0.0**	**1.6 ± 0.0**	**1.8 ± 0.0**	**1.7 ± 0.0**	**1.3 ± 0.0**	**1.2 ± 0.0**	**1.4 ± 0.0**
1110	1112	(*E*)-2, 4-Dimethylhepta-2, 4-dienal	---	---	tr	---	---	tr	tr	---	---	---
1116	1112	*trans*-Thujone	0.6 ± 0.0	0.3 ± 0.0	0.4 ± 0.0	0.7 ± 0.0	0.9 ± 0.0	0.8 ± 0.0	0.5 ± 0.0	0.7 ± 0.0	0.6 ± 0.0	0.6 ± 0.0
1117	1119	Myrcenol	---	---	---	---	---	---	0.1 ± 0.0	---	---	tr
1117	1118	*exo*-Fenchol	---	---	---	---	---	---	---	0.1 ± 0.0	tr	---
1117	1119	*trans-p*-Mentha-2, 8-dien-1-ol	tr	---	---	---	---	---	---	---	---	---
1119	1124	Chrysanthenone	0.2 ± 0.0	0.1 ± 0.0	0.3 ± 0.0	0.4 ± 0.0	0.3 ± 0.0	0.2 ± 0.0	0.4 ± 0.0	0.3 ± 0.0	0.2 ± 0.0	0.2 ± 0.0
1122	1118	*cis-p*-Menth-2-en-1-ol	---	---	---	---	---	---	---	---	---	tr
**1125**	**1122**	**α-Campholenal**	**2.0 ± 0.0**	**1.1 ± 0.0**	**1.7 ± 0.0**	**2.3 ± 0.0**	**1.9 ± 0.0**	**2.8 ± 0.1**	**2.1 ± 0.0**	**2.2 ± 0.0**	**1.8 ± 0.0**	**1.8 ± 0.0**
1130	1132	*cis*-Limonene oxide	---	---	---	---	---	---	---	---	---	---
1135	1137	*trans*-Limonene oxide	---	---	---	---	---	---	---	---	---	---
1137	1137	*trans*-Sabinol	---	---	tr	---	---	0.1 ± 0.0	---	---	---	---
**1139**	**1135**	***trans*-Pinocarveol**	**2.4 ± 0.0**	**1.3 ± 0.0**	**1.6 ± 0.0**	**2.7 ± 0.0**	**2.0 ± 0.1**	**2.8 ± 0.0**	**2.5 ± 0.0**	**2.3 ± 0.0**	**1.9 ± 0.0**	**1.3 ± 0.0**
1139	1137	*cis*-Verbenol	0.6 ± 0.0	0.2 ± 0.0	0.2 ± 0.0	1.2 ± 0.0	0.2 ± 0.0	0.5 ± 0.0	0.4 ± 0.0	0.5 ± 0.0	0.2 ± 0.0	tr
**1143**	**1140**	***trans*-Verbenol**	**1.6 ± 0.0**	**1.4 ± 0.0**	**0.9 ± 0.0**	**2.9 ± 0.3**	**1.6 ± 0.0**	**2.2 ± 0.0**	**1.9 ± 0.0**	**2.8 ± 0.0**	**1.2 ± 0.0**	**0.6 ± 0.0**
1145	1141	Camphor	0.2 ± 0.0	tr	0.2 ± 0.0	0.1 ± 0.0	0.2 ± 0.0	0.3 ± 0.0	0.3 ± 0.0	0.2 ± 0.0	0.3 ± 0.0	0.2 ± 0.0
1148	1150	α-Phellandren-8-ol	0.7 ± 0.0	0.2 ± 0.0	0.5 ± 0.0	0.3 ± 0.0	0.7 ± 0.0	0.8 ± 0.0	0.5 ± 0.0	0.7 ± 0.0	0.8 ± 0.0	0.5 ± 0.0
1158	1158	*trans*-Pinocamphone	0.5 ± 0.0	0.4 ± 0.0	0.6 ± 0.0	0.8 ± 0.0	0.4 ± 0.0	0.8 ± 0.0	0.5 ± 0.0	0.5 ± 0.0	0.5 ± 0.0	0.5 ± 0.0
1160	1160	Pinocarvone	0.5 ± 0.0	0.2 ± 0.0	0.4 ± 0.0	0.3 ± 0.0	0.4 ± 0.0	0.5 ± 0.0	0.5 ± 0.0	0.5 ± 0.0	0.3 ± 0.0	0.4 ± 0.0
1167	1168	*trans*-Phellandrene epoxide	---	---	0.1 ± 0.0	---	---	0.1 ± 0.0	---	tr	tr	tr
**1169**	**1166**	***p*-Mentha-1, 5-dien-8-ol**	**1.5 ± 0.0**	**0.5 ± 0.0**	**1.2 ± 0.0**	**1.3 ± 0.0**	**0.9 ± 0.0**	**1.8 ± 0.0**	**1.1 ± 0.0**	**1.1 ± 0.038**	**1.3 ± 0.0**	**1.0 ± 0.0**
1170	1165	Borneol	---	---	---	---	---	---	---	---	---	tr
1174	1172	*cis*-Pinocamphone	tr	tr	tr	tr	tr	0.1 ± 0.0	tr	0.1 ± 0.0	tr	---
1178	1174	Terpinen-4-ol	0.4 ± 0.0	0.1 ± 0.0	0.3 ± 0.0	0.4 ± 0.0	0.4 ± 0.0	0.3 ± 0.0	0.3 ± 0.0	0.3 ± 0.0	0.4 ± 0.0	0.2 ± 0.0
1185	1179	*p*-Cymen-8-ol	0.8 ± 0.0	0.5 ± 0.0	0.8 ± 0.0	0.8 ± 0.0	0.7 ± 0.0	1.4 ± 0.0	0.7 ± 0.0	0.8 ± 0.0	0.9 ± 0.0	0.7 ± 0.0
1192	1186	α-Terpineol	1.2 ± 0.0	0.5 ± 0.0	0.7 ± 0.0	1.1 ± 0.0	0.9 ± 0.0	1.4 ± 0.0	0.9 ± 0.0	1.2 ± 0.0	1.0 ± 0.0	0.9 ± 0.0
**1193**	**1195**	**Myrtenal**	**1.8 ± 0.0**	**1.1 ± 0.0**	**1.6 ± 0.0**	**2.1 ± 0.0**	**1.5 ± 0.0**	**1.8 ± 0.0**	**1.6 ± 0.0**	**1.6 ± 0.0**	**1.7 ± 0.0**	**1.1 ± 0.0**
**1205**	**1204**	**Verbenone**	**2.8 ± 0.0**	**1.2 ± 0.0**	**2.0 ± 0.0**	**3.0 ± 0.0**	**2.2 ± 0.0**	**2.9 ± 0.0**	**2.4 ± 0.0**	**2.7 ± 0.0**	**2.5 ± 0.0**	**2.1 ± 0.0**
1216	1215	*trans*-Carveol	0.1	tr	---	---	---	tr	tr	0.2 ± 0.0	tr	---
**1242**	**1239**	**Carvone**	**5.1 ± 0.1**	**2.1 ± 0.1**	**4.3 ± 0.0**	**4.4 ± 0.2**	**4.2 ± 0.1**	**5.4 ± 0.1**	**4.6 ± 0.1**	**4.7 ± 0.0**	**5.0 ± 0.0**	**4.3 ± 0.1**
1246	1254	Linalyl acetate	---	---	tr	---	---	tr	tr	tr	tr	---
1261	1265	3, 5-Dimethoxytoluene	---	tr	tr	---	---	tr	tr	tr	---	tr
**1281**	**1287**	**Bornyl acetate**	**3.4 ± 0.2**	**1.6 ± 0.0**	**2.9 ± 0.0**	**3.5 ± 0.3**	**2.6 ± 0.1**	**3.5 ± 0.1**	**2.6 ± 0.0**	**3.0 ± 0.0**	**2.9 ± 0.0**	**2.6 ± 0.0**
1286	1289	Thymol	---	---	tr	---	---	tr	---	---	---	tr
**1294**	**1298**	**Carvacrol**	**2.2 ± 0.0**	**0.9 ± 0.0**	**1.6 ± 0.0**	**2.1 ± 0.0**	**1.7 ± 0.0**	**1.9 ± 0.0**	**1.7 ± 0.0**	**1.8 ± 0.0**	**1.8 ± 0.0**	**1.6 ± 0.0**
**1343**	**1346**	**α-Terpinyl acetate**	**2.1 ± 0.0**	**0.8 ± 0.0**	**1.5 ± 0.0**	**2.1 ± 0.0**	**1.6 ± 0.0**	**2.5 ± 0.0**	**1.7 ± 0.0**	**1.6 ± 0.0**	**1.9 ± 0.0**	**1.6 ± 0.0**
**1345**	**1345**	**α-Cubebene**	**3.4 ± 0.0**	**1.2 ± 0.0**	**2.7 ± 0.0**	**2.7 ± 0.1**	**2.6 ± 0.0**	**2.8 ± 0.0**	**2.4 ± 0.0**	**2.5 ± 0.0**	**2.7 ± 0.0**	**2.4 ± 0.0**
**1373**	**1374**	**α-Copaene**	**4.8 ± 0.4**	**1.8 ± 0.0**	**3.9 ± 0.0**	**4.7 ± 0.1**	**3.8 ± 0.1**	**5.0 ± 0.1**	**4.1 ± 0.1**	**3.6 ± 0.0**	**4.1 ± 0.0**	**3.8 ± 0.0**
1409	1411	*cis*-α-Bergamotene	---	tr	tr	---	---	---	tr	tr	tr	tr
1416	1417	β-Caryophyllene	---	---	---	---	---	---	tr	---	---	tr
1429	1432	*trans*-α-Bergamotene	---	tr	tr	---	---	---	tr	tr	0.2 ± 0.0	tr
1441	1440	(*Z*)-β-Farnesene	---	---	---	---	---	---	---	---	---	tr
1441	1449	α-Himachalene	---	---	---	---	---	---	---	---	---	---
1452	1452	α-Humulene	---	---	---	---	---	---	---	---	---	---
1486	1489	β-Selinene	---	---	---	---	---	---	tr	---	---	tr
1493	1498	α-Selinene	---	---	---	---	---	---	---	---	---	---
1579	1582	Caryophyllene oxide	---	---	---	---	---	---	tr	tr	---	tr
1942	1944	*m*-Camphorene	0.1 ± 0.0	0.2 ± 0.0	---	---	0.1 ± 0.0	---	0.1 ± 0.0	---	0.1 ± 0.0	0.2 ± 0.0
1948	1947	(3*E*)-Cembrene A	0.1 ± 0.0	0.4 ± 0.0	0.1 ± 0.0	0.7 ± 0.0	0.1 ± 0.0	0.2 ± 0.0	0.4 ± 0.0	0.2 ± 0.0	0.2 ± 0.0	0.4 ± 0.0
1977	1977	*p*-Camphorene	tr	tr	---	---	tr	---	0.2 ± 0.0	---	---	tr
1993	1992	α-Pinacene	---	tr	tr	---	---	---	tr	tr	tr	tr
2130	2138	Cembrenol	0.1 ± 0.0	0.4 ± 0.0	0.1 ± 0.0	0.5 ± 0.0	0.1 ± 0.0	0.3 ± 0.0	0.4 ± 0.0	0.3 ± 0.0	0.1 ± 0.0	0.2 ± 0.0
2143	2144	Incensole + Serratol	0.4 ± 0.0	0.9 ± 0.0	0.4 ± 0.0	0.5 ± 0.0	0.3 ± 0.0	0.9 ± 0.0	0.4 ± 0.0	0.2 ± 0.0	0.2 ± 0.0	0.8 ± 0.0
		Monoterpene hydrocarbons	56.6	76.4	65.1	52.5	62.4	49.6	60.5	59.9	61.5	65.9
		Oxygenated monoterpenoids	33.9	17.8	27.0	36.7	29.6	39.8	31.2	33.1	30.5	26.1
		Sesquiterpenoids	8.2	3.0	6.7	7.5	6.4	7.7	6.5	6.1	7.0	6.3
		Diterpenoids	0.7	1.8	0.7	1.8	0.6	1.3	1.4	0.6	0.6	1.6
		Others	0.1	0.1	0.4	0.5	0.3	0.5	0.1	0.2	0.0	0.0
		Total Identified	99.6	99.1	99.8	99.0	99.3	99.0	99.8	99.9	99.5	99.9
**RI a**	**RI b**	**Compound**	**Re190227G**	**Re190227H**	**Re190227I**	**Re190227K**	**Re190227L**	**Re190227N**	**Re190227O**	**Re190227P**	**Re190416A**	**Re190416B**
**906**	**906**	**Santolina triene c**	**1.2 ± 0.0 d**	**1.4 ± 0.0**	**1.0 ± 0.0**	**1.2 ± 0.0**	**1.1 ± 0.0**	**1.4 ± 0.0**	**0.9 ± 0.0**	**1.3 ± 0.0**	**0.9 ± 0.0**	**0.9 ± 0.0**
**918**	**919**	**5, 5-Dimethyl-1-vinylbicyclo [2.1.1] hexane**	**0.8 ± 0.0**	**0.9 ± 0.0**	**1.4 ± 0.0**	**1.3 ± 0.0**	**0.8 ± 0.0**	**1.4 ± 0.0**	**1.2 ± 0.0**	**0.8 ± 0.0**	**0.6 ± 0.0**	**0.6 ± 0.0**
920	921	Tricyclene	0.2 ± 0.0	0.2 ± 0.0	0.1 ± 0.0	0.1 ± 0.0	0.2 ± 0.0	0.1 ± 0.0	0.2 ± 0.0	0.2 ± 0.0	0.1 ± 0.0	0.2 ± 0.0
**923**	**924**	**α-Thujene**	**1.4 ± 0.0**	**2.7 ± 0.0**	**0.8 ± 0.0**	**3.4 ± 0.0**	**0.9 ± 0.0**	**2.3 ± 0.0**	**0.5 ± 0.0**	**2.0 ± 0.0**	**2.0 ± 0.0**	**2.1 ± 0.0**
**932**	**932**	**α-Pinene**	**51.0 ± 0.2**	**41.8 ± 0.3**	**44.9 ± 0.4**	**39.6 ± 0.3**	**38.2 ± 0.3**	**47.1 ± 0.5**	**40.3 ± 0.3**	**42.0 ± 0.3**	**55.2 ± 0.4**	**54.2 ± 0.2**
945	945	α-Fenchene	tr e	tr	tr	tr	tr	tr	tr	tr	tr	tr
**947**	**946**	**Camphene**	**0.8 ± 0.0**	**1.1 ± 0.0**	**1.0 ± 0.0**	**1.0 ± 0.0**	**0.9 ± 0.0**	**0.8 ± 0.0**	**0.8 ± 0.0**	**0.8 ± 0.0**	**1.0 ± 0.0**	**1.1 ± 0.0**
951	953	Thuja-2, 4(10)-diene	0.6 ± 0.0	0.9 ± 0.0	0.7 ± 0.0	0.6 ± 0.0	0.6 ± 0.0	0.4 ± 0.0	0.6 ± 0.0	0.8 ± 0.0	0.7 ± 0.0	0.7 ± 0.0
953	954	β-Fenchene	0.1 ± 0.0	0.1 ± 0.0	0.5 ± 0.0	0.4 ± 0.0	0.2 ± 0.0	0.1 ± 0.0	0.1 ± 0.0	0.1 ± 0.0	0.5 ± 0.0	0.3 ± 0.0
**970**	**969**	**Sabinene**	**0.7 ± 0.0**	**1.4 ± 0.0**	**0.8 ± 0.0**	**1.0 ± 0.0**	**1.0 ± 0.0**	**1.3 ± 0.0**	**1.1 ± 0.0**	**1.0 ± 0.0**	**1.1 ± 0.0**	**1.1 ± 0.0**
**975**	**974**	**β-Pinene**	**1.6 ± 0.0**	**2.6 ± 0.0**	**1.8 ± 0.0**	**1.5 ± 0.0**	**1.2 ± 0.0**	**1.4 ± 0.0**	**1.4 ± 0.0**	**1.1 ± 0.0**	**2.0 ± 0.0**	**1.9 ± 0.0**
985	984	*trans-p*-Mentha-2, 8-diene	---	---	tr	---	tr	tr	---	---	tr	tr
**986**	**988**	**Myrcene**	**0.6 ± 0.0**	**0.5 ± 0.0**	**1.9 ± 0.0**	**3.7 ± 0.0**	**0.8 ± 0.0**	**5.5 ± 0.1**	**2.7 ± 0.0**	**0.7 ± 0.0**	**0.5 ± 0.0**	**0.7 ± 0.0**
988	989	3, 3, 7-Trimethylcyclohepta-1, 3, 5-triene	---	0.1 ± 0.0	tr	tr	---	---	0.1 ± 0.0	---	tr	tr
996	997	(*E*)-2, 6-Dimethyl-2, 6-octadiene	---	---	tr	tr	---	---	---	---	0.1 ± 0.0	tr
998	1001	δ-2-Carene	---	---	tr	tr	---	---	---	---	tr	tr
999	995	*cis-p*-Menth-8-ene	0.5 ± 0.0	0.5 ± 0.0	0.7 ± 0.0	0.6 ± 0.0	0.7 ± 0.0	0.7 ± 0.0	0.4 ± 0.0	0.5 ± 0.0	0.3 ± 0.0	---
1002	1003	*p*-Mentha-1(7), 8-diene	---	---	---	---	---	---	---	---	---	---
1003	1002	α-Phellandrene	tr	tr	0.1 ± 0.0	tr	tr	0.2 ± 0.0	0.1 ± 0.0	0.1 ± 0.0	0.1 ± 0.0	0.1 ± 0.0
1004	1006	1,5,8-*p*-Menthatriene	0.3 ± 0.0	0.2 ± 0.0	0.3 ± 0.0	0.4 ± 0.0	0.5 ± 0.0	0.4 ± 0.0	0.6 ± 0.0	0.5 ± 0.0	---	0.3 ± 0.0
1006	1005	*o*-Cresol methyl ether	0.1 ± 0.0	tr	0.1 ± 0.0	tr	0.1 ± 0.0	tr	0.3 ± 0.0	0.1 ± 0.0	---	0.1 ± 0.0
1006	1008	δ-3-Carene	tr	tr	0.1 ± 0.0	tr	tr	tr	tr	tr	tr	0.1 ± 0.0
1015	1014	α-Terpinene	0.3 ± 0.0	0.2 ± 0.0	0.4 ± 0.0	0.6 ± 0.0	0.5 ± 0.0	0.5 ± 0.0	0.4 ± 0.0	0.3 ± 0.0	0.3 ± 0.0	0.4 ± 0.0
1017	1022	*m*-Cymene	0.5 ± 0.0	0.5 ± 0.0	0.5 ± 0.0	0.6 ± 0.0	0.5 ± 0.0	0.6 ± 0.0	0.6 ± 0.0	0.4 ± 0.0	0.5 ± 0.0	0.4 ± 0.0
1021	1021	*p*-Menth-1-ene	---	---	---	---	---	---	0.1 ± 0.0	---	0.6 ± 0.0	0.6 ± 0.0
1022	1024	*p*-Cymene	0.5 ± 0.0	0.6 ± 0.0	0.5 ± 0.0	0.6 ± 0.0	0.6 ± 0.0	0.4 ± 0.0	0.8 ± 0.0	0.4 ± 0.0	0.5 ± 0.0	0.5 ± 0.0
1024	1026	2-Acetyl-5-methylfuran	---	0.2 ± 0.0	tr	0.1 ± 0.0	---	tr	tr	---	0.1 ± 0.0	tr
1027	1024	Limonene	tr	0.1 ± 0.0	0.1 ± 0.0	0.1 ± 0.0	0.1 ± 0.0	0.1 ± 0.0	0.1 ± 0.0	0.1 ± 0.0	0.1 ± 0.0	0.1 ± 0.0
1028	1025	β-Phellandrene	0.4 ± 0.0	---	0.1 ± 0.0	tr	0.1 ± 0.0	0.2 ± 0.0	tr	0.3 ± 0.0	tr	tr
**1029**	**1026**	**1,8-Cineole**	**1.3 ± 0.0**	**3. ± 0.0**	**2.1 ± 0.0**	**4.6 ± 0.1**	**5.1 ± 0.0**	**1.3 ± 0.0**	**1.2 ± 0.0**	**6.1 ± 0.1**	**1.4 ± 0.0**	**1.3 ± 0.0**
1031	1032	(*Z*)-β-Ocimene	0.1 ± 0.0	0.3 ± 0.0	0.1 ± 0.0	0.1 ± 0.0	0.2 ± 0.0	0.1 ± 0.0	0.1 ± 0.0	0.2 ± 0.0	0.5 ± 0.0	0.1 ± 0.0
**1032**	**1039**	***o*-Cymene**	**1.3 ± 0.0**	**1.2 ± 0.0**	**1.4 ± 0.0**	**1.2 ± 0.0**	**1.3 ± 0.0**	**1.4 ± 0.0**	**1.4 ± 0.0**	**1.0 ± 0.0**	**0.7 ± 0.0**	**1.1 ± 0.0**
1043	1044	(*E*)-β-Ocimene	0.1 ± 0.0	tr	0.6 ± 0.0	0.2	0.5 ± 0.0	0.1 ± 0.0	0.1 ± 0.0	0.5 ± 0.0	0.2 ± 0.0	0.2 ± 0.0
**1055**	**1054**	**γ-Terpinene**	**1.5 ± 0.0**	**1.8 ± 0.0**	**1.8 ± 0.0**	**2.0 ± 0.0**	**2.0 ± 0.0**	**1.5 ± 0.0**	**2.4 ± 0.0**	**1.9 ± 0.0**	**1.5 ± 0.0**	**1.4 ± 0.0**
1068	1065	*cis*-Sabinene hydrate	---	tr	tr	tr	---	---	tr	tr	tr	tr
**1083**	**1086**	**Terpinolene**	**1.1 ± 0.0**	**1.0 ± 0.0**	**1.3 ± 0.0**	**1.3 ± 0.0**	**1.2 ± 0.0**	**1.0 ± 0.0**	**1.5 ± 0.0**	**0.9 ± 0.0**	**1.0 ± 0.0**	**1.1 ± 0.0**
1088	1089	*p*-Cymenene	---	---	---	---	---	---	---	---	---	---
1088	1090	6,7-Epoxymyrcene	tr	---	---	---	---	---	---	tr	---	---
1090	1095	6-Camphenone	---	---	---	---	---	---	---	---	0.1 ± 0.0	0.1 ± 0.0
1094	1091	Rosefuran	---	---	---	---	---	---	---	---	---	---
1096	1102	Perillene	0.1 ± 0.0	---	---	---	0.1 ± 0.0	0.1 ± 0.0	tr	tr	---	---
1097	1095	Linalool	0.8 ± 0.0	1.3 ± 0.0	0.9 ± 0.0	1.1 ± 0.0	1.4 ± 0.0	0.9 ± 0.0	1.1 ± 0.0	1.2 ± 0.0	tr	---
1097	1099	α-Pinene oxide	---	tr	tr	---	0.1 ± 0.0	---	tr	---	---	0.1 ± 0.0
1099	1098	*trans*-Sabinene hydrate	---	tr	---	---	---	---	---	tr	0.6 ± 0.0	0.6 ± 0.0
1103	1101	*cis*-Thujone	1.3 ± 0.0	1.2 ± 0.0	1.3 ± 0.0 ± 0.0	1.3 ± 0.0	1.3 ± 0.0	1.2 ± 0.0	1.6 ± 0.0	0.9 ± 0.0	---	---
1110	1112	(*E*)-2, 4-Dimethylhepta-2, 4-dienal	---	tr	---	---	---	---	---	---	tr	---
1116	1112	*trans*-Thujone	0.4 ± 0.0	0.5 ± 0.0	0.6 ± 0.0	0.6 ± 0.0	0.7 ± 0.0	0.5 ± 0.0	1.0 ± 0.0	0.4 ± 0.0	0.4 ± 0.0	0.5 ± 0.0
1117	1119	Myrcenol	---	---	---	---	---	---	---	---	---	---
1117	1118	*exo*-Fenchol	---	---	---	---	---	---	---	---	---	---
1117	1119	*trans-p*-Mentha-2, 8-dien-1-ol	---	---	---	---	---	---	---	---	---	---
1119	1124	Chrysanthenone	0.2 ± 0.0	0.3 ± 0.0	0.2 ± 0.0	0.2 ± 0.0	0.3 ± 0.0	0.2 ± 0.0	0.3 ± 0.0	0.2 ± 0.0	0.1 ± 0.0	0.2 ± 0.0
1122	1118	*cis-p*-Menth-2-en-1-ol	---	---	---	---	---	---	---	---	---	---
**1125**	**1122**	**α-Campholenal**	**1.6 ± 0.0**	**1.7 ± 0.0**	**1.4 ± 0.0**	**1.6 ± 0.0**	**2.1 ± 0.0**	**1.4 ± 0.0**	**1.8 ± 0.0**	**1.7 ± 0.0**	**1.5 ± 0.0**	**1.5 ± 0.0**
1130	1132	*cis*-Limonene oxide	---	0.1 ± 0.0	---	tr	tr	tr	0.1 ± 0.0	---	tr	tr
1135	1137	*trans*-Limonene oxide	---	0.2 ± 0.0	tr	tr	tr	tr	0.1 ± 0.0	---	---	---
1137	1137	*trans*-Sabinol	---	---	---	---	---	---	---	---	---	---
**1139**	**1135**	***trans*-Pinocarveol**	**1.4 ± 0.0**	**2.1 ± 0.0**	**1.7 ± 0.0**	**1.7 ± 0.0**	**1.3 ± 0.0**	**1.5 ± 0.0**	**1.9 ± 0.0**	**2.5 ± 0.0**	**2.0 ± 0.0**	**1.6 ± 0.0**
1139	1137	*cis*-Verbenol	tr	0.7 ± 0.0	tr	0.3 ± 0.0	tr	tr	0.6 ± 0.0	0.1 ± 0.0	0.4 ± 0.0	0.2 ± 0.0
**1143**	**1140**	***trans*-Verbenol**	**0.7 ± 0.0**	**1.3 ± 0.0**	**0.5 ± 0.0**	**0.6 ± 0.0**	**1.7 ± 0.0**	**0.8 ± 0.0**	**0.7 ± 0.0**	**2.0 ± 0.0**	**2.0 ± 0.1**	**1.3 ± 0.0**
1145	1141	Camphor	0.2 ± 0.0	0.2 ± 0.0	0.1 ± 0.0	0.3 ± 0.0	0.2 ± 0.0	0.1 ± 0.0	0.3 ± 0.0	0.2 ± 0.0	0.1 ± 0.0	0.1 ± 0.0
1148	1150	α-Phellandren-8-ol	0.5 ± 0.0	0.7 ± 0.0	0.5 ± 0.0	0.5 ± 0.0	0.7 ± 0.0	0.5 ± 0.0	0.7 ± 0.0	0.7 ± 0.0	0.5 ± 0.0	0.5 ± 0.0
1158	1158	*trans*-Pinocamphone	0.4 ± 0.0	0.5 ± 0.0	0.6 ± 0.0	0.8 ± 0.0	0.6 ± 0.0	0.4 ± 0.0	0.7 ± 0.0	0.6 ± 0.0	0.5 ± 0.0	0.5 ± 0.0
1160	1160	Pinocarvone	0.3 ± 0.0	0.4 ± 0.0	0.3 ± 0.0	0.3 ± 0.0	0.5 ± 0.0	0.3 ± 0.0	0.5 ± 0.0	0.4 ± 0.0	0.2 ± 0.0	0.2 ± 0.0
1167	1168	*trans*-Phellandrene epoxide	---	tr	tr	tr	---	---	---	---	tr	tr
**1169**	**1166**	***p*-Mentha-1,5-dien-8-ol**	**1.1 ± 0.0**	**1.3 ± 0.0**	**1.0 ± 0.0**	**1.1 ± 0.0**	**1.4 ± 0.0**	**1.1 ± 0.0**	**1.3 ± 0.0**	**1.4 ± 0.0**	**1.0 ± 0.0**	**0.9 ± 0.0**
1170	1165	Borneol	---	---	---	---	---	---	---	tr	---	---
1174	1172	*cis*-Pinocamphone	tr	tr	tr	tr	tr	tr	tr	tr	tr	tr
1178	1174	Terpinen-4-ol	0.4 ± 0.0	0.4 ± 0.0	0.3 ± 0.0	0.2 ± 0.0	0.3 ± 0.0	0.3 ± 0.0	0.4 ± 0.0	0.2 ± 0.0	0.3 ± 0.0	0.2 ± 0.0
1185	1179	*p*-Cymen-8-ol	0.5 ± 0.0	0.8 ± 0.0	0.7 ± 0.0	0.7 ± 0.0	0.9 ± 0.0	0.6 ± 0.0	1.0 ± 0.0	0.8 ± 0.0	0.6 ± 0.0	0.6 ± 0.0
1192	1186	α-Terpineol	0.7 ± 0.0	0.8 ± 0.0	0.9 ± 0.0	1.1 ± 0.0	1.0 ± 0.0	0.8 ± 0.0	1.0 ± 0.0	1.0 ± 0.0	0.7 ± 0.0	0.6 ± 0.0
**1193**	**1195**	**Myrtenal**	**1.2 ± 0.0**	**1.5 ± 0.0**	**1.2 ± 0.0**	**1.2 ± 0.0**	**1.7 ± 0.0**	**1.2 ± 0.0**	**1.5 ± 0.0**	**1.7 ± 0.0**	**1.3 ± 0.0**	**1.2 ± 0.0**
**1205**	**1204**	**Verbenone**	**1.9 ± 0.0**	**2.3 ± 0.0**	**2.2 ± 0.0**	**2.0 ± 0.0**	**2.7 ± 0.0**	**1.7 ± 0.0**	**2.6 ± 0.0**	**2.2 ± 0.0**	**1.8 ± 0.0**	**1.8 ± 0.0**
1216	1215	*trans*-Carveol	---	0.3 ± 0.0	tr	0.1	---	---	0.2 ± 0.0	---	0.2 ± 0.0	0.1 ± 0.0
**1242**	**1239**	**Carvone**	**4.2 ± 0.1**	**4.2 ± 0.0**	**4.3 ± 0.1**	**4.4 ± 0.1**	**3.3 ± 0.0**	**3.6 ± 0.0**	**5.1 ± 0.1**	**4.4 ± 0.0**	**3.7 ± 0.1**	**3.4 ± 0.3**
1246	1254	Linalyl acetate	---	---	---	---	---	---	---	---	---	---
1261	1265	3,5-Dimethoxytoluene	tr	---	---	---	---	---	---	---	---	---
**1281**	**1287**	**Bornyl acetate**	**2.4 ± 0.0**	**2.8 ± 0.0**	**2.7 ± 0.0**	**2.6 ± 0.0**	**3.0 ± 0.0**	**2.4 ± 0.0**	**3.1 ± 0.0**	**2.4 ± 0.0**	**2.3 ± 0.0**	**2.4 ± 0.0**
1286	1289	Thymol	---	tr	---	---	---	---	---	---	---	---
**1294**	**1298**	**Carvacrol**	**1.7 ± 0.0**	**1.7 ± 0.0**	**1.9 ± 0.0**	**1.6 ± 0.0**	**1.9 ± 0.0**	**1.3 ± 0.0**	**2.0 ± 0.0**	**1.9 ± 0.0**	**---**	**---**
**1343**	**1346**	**α-Terpinyl acetate**	**1.9 ± 0.0**	**1.6 ± 0.0**	**1.8 ± 0.0**	**1.6 ± 0.0**	**1.4 ± 0.0**	**1.4 ± 0.0**	**2.0 ± 0.0**	**1.7 ± 0.0**	**1.3 ± 0.0**	**1.5 ± 0.0**
**1345**	**1345**	**α-Cubebene**	**2.6 ± 0.0**	**2.2 ± 0.0**	**2.5 ± 0.0**	**2.4 ± 0.0**	**2.9 ± 0.0**	**2.2 ± 0.0**	**2.9 ± 0.0**	**2.5 ± 0.0**	**2.1 ± 0.0**	**2.0 ± 0.0**
**1373**	**1374**	**α-Copaene**	**3.8 ± 0.0**	**3.5 ± 0.0**	**3.7 ± 0.1**	**3.6 ± 0.1**	**2.9 ± 0.0**	**3.2 ± 0.0**	**4.1 ± 0.0**	**3.7 ± 0.0**	**2.5 ± 0.0**	**3.5 ± 0.1**
1409	1411	*cis*-α-Bergamotene	tr	tr	tr	tr	0.2 ± 0.0	tr	tr	tr	tr	0.1 ± 0.0
1416	1417	β-Caryophyllene	tr	tr	tr	tr	tr	tr	tr	tr	---	tr
1429	1432	*trans*-α-Bergamotene	tr	tr	tr	tr	0.2 ± 0.0	tr	tr	tr	tr	0.1 ± 0.0
1441	1440	(*Z*)-β-Farnesene	---	---	---	---	---	---	---	---	---	---
1441	1449	α-Himachalene	---	---	---	---	tr	tr	---	---	---	---
1452	1452	α-Humulene	tr	---	---	---	tr	---	---	---	---	---
1486	1489	β-Selinene	tr	---	---	tr	tr	---	---	---	---	---
1493	1498	α-Selinene	tr	---	---	---	tr	---	---	---	---	---
1579	1582	Caryophyllene oxide	tr	tr	---	---	tr	tr	tr	tr	---	---
1942	1944	*m*-Camphorene	0.3 ± 0.0	---	1.8 ± 0.0	0.8 ± 0.0	1.8 ± 0.0	0.2 ± 0.0	0.2 ± 0.0	tr	---	---
1948	1947	(3E)-Cembrene A	0.7 ± 0.0	0.2 ± 0.0	0.5 ± 0.0	0.1 ± 0.0	0.9 ± 0.0	0.4 ± 0.0	0.2 ± 0.0	0.4 ± 0.0	0.1 ± 0.0	0.2 ± 0.0
1977	1977	*p*-Camphorene	0.1 ± 0.0	---	---	---	2.1 ± 0.0	0.6 ± 0.0	tr	tr	---	---
1993	1992	α-Pinacene	tr	tr	tr	---	0.1 ± 0.0	tr	tr	tr	---	0.1 ± 0.0
2130	2138	Cembrenol	0.4 ± 0.0	0.1 ± 0.0	0.8 ± 0.0	tr	0.5 ± 0.0	0.2 ± 0.0	0.2 ± 0.0	0.1 ± 0.0	0.2 ± 0.0	0.6 ± 0.0
2143	2144	Incensole + Serratol	0.7 ± 0.0	0.6 ± 0.0	0.6 ± 0.0	0.3 ± 0.0	0.2 ± 0.0	0.1 ± 0.0	0.3 ± 0.0	0.5 ± 0.0	0.3 ± 0.0	1.0 ± 0.0
		Monoterpene hydrocarbons	65.7	60.1	62.7	61.5	53.8	69.2	58.5	57.9	70.9	70.0
		Oxygenated monoterpenoids	25.1	32.5	27.1	30.5	33.8	23.5	33.0	34.6	23.0	21.4
		Sesquiterpenoids	6.4	5.7	6.2	5.9	6.1	5.4	7.0	6.1	4.6	5.7
		Diterpenoids	2.3	0.9	3.7	1.1	5.6	1.6	0.9	1.0	0.6	1.9
		Others	0.1	0.2	0.1	0.1	0.1	0.0	0.4	0.1	0.1	0.1
		Total Identified	99.6	99.4	99.8	99.1	99.4	99.6	99.8	99.7	99.2	99.0

^a^ RI: Retention Index determined in reference to a homologous series of *n*-alkanes on a ZB-5ms column. ^b^ RI: Retention Index from the databases. ^c^ Entries in boldface were used in the cluster analysis. ^d^ Percentages are average of three runs (±standard deviations). ^e^ tr = “trace” (<0.05%).

**Table 2:** Geographical collection locations of *Boswellia dalzielii* oleogum resins.

Sample Code	GPS Coordinates		Elevation, m
Re190227M	12°21′13.92″ N	3°16′57.72″ W	290
Re190125O	12°41′43.74″ N	1°10′48.18″ W	291
Re190125P	12°41′43.74″ N	1°10′48.18″ W	291
Re190125Q	12°41′43.74″ N	1°10′48.18″ W	291
Re190125R	12°41′43.74″ N	1°10′48.18″ W	291
Re190125S	12°41′43.74″ N	1°10′48.18″ W	291
Re190125T	12°29′36.54″ N	1°15′40.20″ W	321
Re190125U	12°29′36.54″ N	1°15′40.20″ W	321
Re190125V	12°29′36.54″ N	1°15′40.20″ W	321
Re190125W	12°21′28.98″ N	1°3′15.60″ W	284
Re190125X	12°20′5.58″ N	0°59′10.14″ W	318
Re190227G	12°21′13.92″ N	3°16′57.72″ W	290
Re190227H	12°21′13.92″ N	3°16′57.72″ W	290
Re190227I	12°21′13.92″ N	3°16′57.72″ W	290
Re190227K	12°21′13.92″ N	3°16′57.72″ W	290
Re190227L	12°21′13.92″ N	3°16′57.72″ W	290
Re190227N	12°21′13.92″ N	3°16′57.72″ W	290
Re190227P	12°21′13.92″ N	3°16′57.72″ W	290
Re190416A	12°46′53.88″ N	1°19′20.16″ W	348
Re190416B	12°45′56.70″ N	1°54′19.56″ W	372
